# Supplementary material for: The microbiome biomarkers of pregnant women’s vaginal area predict preterm prelabor rupture in Western China
Source: Front Cell Infect Microbiol. 2024 Oct 31;14:1471027. doi: 10.3389/fcimb.2024.1471027 (PMC11560878; doi:10.3389/fcimb.2024.1471027)
Supplement: Supplementary file 1 [file DataSheet1.zip › compare_1/Community/Pieplot/species/sample/C25.species.pieplot.pdf]

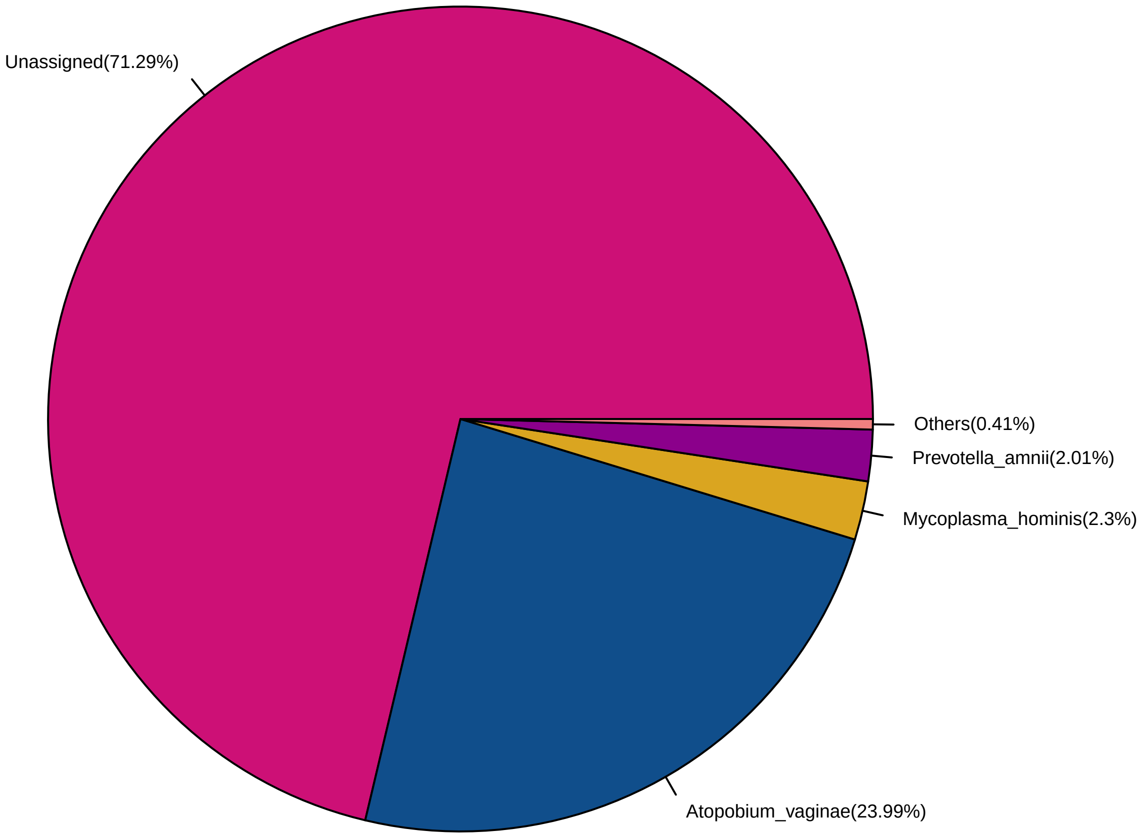

Unassigned(71.29%)
Others(0.41%)
Prevotella_amnii(2.01%)
Mycoplasma_hominis(2.3%)
Atopobium_vaginae(23.99%)
